# A Comprehensive Clinical Review of Maxillary Sinus Floor Elevation in Patients with Well-Defined Faintly Radiopaque Lesions in the Antrum

**DOI:** 10.3390/jcm13020332

**Published:** 2024-01-06

**Authors:** Yasuhiro Nosaka, Hitomi Nosaka, Motohiro Munakata, Minoru Sanda

**Affiliations:** 1 Nosaka Oral Surgery Clinic, Ashiya 659-0083, Japan; nosasen2@gmail.com; 2 Department of Implant Dentistry, Showa University School of Dentistry, Tokyo 145-8515, Japan; munakata@dent.showa-u.ac.jp; 3 Department of Prosthodontics, Showa University School of Dentistry, Tokyo 145-8515, Japan

**Keywords:** sinus floor elevation, pseudocyst, maxillary sinus, β-TCP, endoscope, histology, cone-beam computed tomography

## Abstract

Well-defined, faintly radiopaque lesions are occasionally observed in the antrum of the maxillary sinus in asymptomatic patients during maxillary sinus floor elevation. These lesions are treated as antral pseudocysts (AP) based on the clinical diagnosis in some cases, and maxillary sinus floor elevation is performed without enucleating these lesions. However, further surgery is required after implant placement if the lesion is a mucocele, odontogenic cyst, or tumour. This comprehensive clinical review aimed to identify an appropriate approach for maxillary sinus floor elevation in patients with well-defined, faintly radiopaque lesions in the antrum based on our clinical experience.

## 1. Introduction

Maxillary sinus floor elevation is an established modality for restoring adequate bone volume in the posterior maxilla and facilitating the placement of dental implants. Two methods have been used for maxillary sinus floor elevation: the lateral and crestal approaches [1]. The periosteum underneath the maxillary sinus membrane is elevated in both methods, leading to the creation of sufficient bone volume for implant placement.

Well-defined, faintly radiopaque lesions are incidentally observed in the maxillary sinus cavity of some asymptomatic patients. These lesions are treated similarly to antral pseudocysts (APs) in some cases, and sinus floor elevation is performed without enucleation or pathological diagnosis of the lesions [2,3,4]. AP can migrate upwards following sinus floor elevation. Postoperative swelling of the sinus membrane may occur [5], leading to the development of sinusitis due to the impairment of osteo-meatal complex patency. Additional surgery is required after sinus floor elevation or during the maintenance stage if the lesion is diagnosed as a mucocele [6,7] or tumour [8,9].

Patients are referred to our clinic by their dentists for implant treatment. Panoramic radiography and cone-beam computed tomography (CBCT) are performed during the initial visit, and the maxillary sinuses are examined in detail in patients requiring sinus floor elevation.

This review aimed to evaluate the treatment policies for sinus floor elevation in patients with well-defined, faintly radiopaque lesions in the antrum of the maxillary sinus.

## 2. CT Evaluation

CBCT images of the maxillary sinus are a prerequisite for the examination of lesions and the evaluation of surgical outcomes in patients undergoing sinus floor elevation. Martinez-González reported that CT enabled diagnosis in 100% of the cases, unlike panoramic radiography, which exhibited a diagnostic rate of 28.57% [10]. All patients undergo CBCT evaluations (3D Accuitomo, Morita, Kyoto, Japan; 80 kV, 5.0 mA) before and after sinus floor elevation at our clinic. However, the radiation dose to the patients must always be considered. The radiation dose depends on the CBCT unit, exposure voltage, exposure current, and imaging volume. Lofthag-Hansen reported that the effective dose from 3D Accuitomo, which has a volume size of 60 mm, diameter × 60 mm in length, tube voltage of 75 kV, and tube current of 4.5–5.5 mA, was 52–63 μSv [11]. Li reported the effective dose from 3D Accuitomo as 54 μSv and that of CB MercuRay (Hitachi Medical Systems America, Twinsburg, OH, USA) using a panoramic field of view as 560 μSv [12].

Thus, 3D Accuitomo appears to be safe for post-operative examination of sinus floor elevation.

## 3. Differential Diagnoses of Pseudocyst

AP is the primary consideration for diagnosis when well-defined, faintly radiopaque antral lesions are observed on the CT images of asymptomatic patients. However, in patients undergoing sinus floor elevation, artificial bone or implants are placed close to the lesions. Therefore, accurate diagnosis of well-defined, faintly radiopaque antral lesions is important, and differential diagnoses should be considered before sinus floor elevation.

### 3.1. Periodontitis or Periapical Diseases

The maxillary premolars and molars are located in close proximity to the maxillary sinus floor, and their roots can perforate through the sinus via the pneumatization of the maxillary sinus [13]. Consequently, infection can extend to the maxillary sinus when the remaining teeth have periodontitis or periapical diseases, resulting in the swelling of the sinus membrane. The CT images often reveal mucosal hyperplasia of the sinus membrane or well-defined, faintly radiopaque antral lesions in these situations. Therefore, the diseased teeth should be treated prior to sinus floor elevation.

#### 3.1.1. Case 1

**Figure 1:**
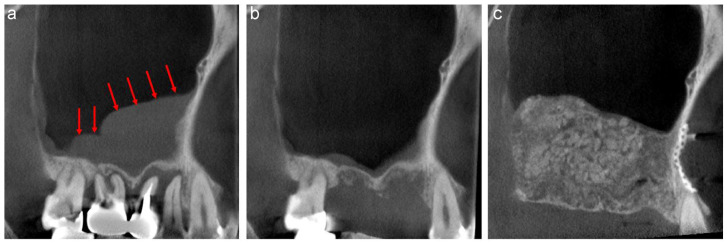
The mesiodistal section of CT images of the right maxillary sinus. (**a**) Antral, well-defined, faintly radiopaque lesions (red arrows) and marked alveolar bone resorption are observed near 16, 15, and 14. (**b**) The well-defined, faintly radiopaque antral lesions disappeared 3 months after extraction. (**c**) A radiopaque area is observed at the sinus floor elevation without swelling of the sinus membrane six months postoperatively.

A 58-year-old male was referred to our clinic by his dentist for implant treatment of the right maxilla in 2018. Well-defined, faintly radiopaque antral lesions were observed on the mesiodistal section of the CT image acquired at the first visit. Moreover, marked alveolar bone resorption was observed in the regions surrounding 16, 15, and 14 (Figure 1a, red arrows). The requirement for extraction of these teeth and sinus floor elevation for implant treatment was explained to the patient. The well-defined, faintly radiopaque antral lesions were found to have disappeared on the CT image acquired 3 months after the extractions (Figure 1b); thus, sinus floor elevation was performed using β-TCP granules (Osferion; Olympus Terumo Biomaterials, Tokyo, Japan) alone and 17 was extracted simultaneously at our clinic. CT images acquired 6 months postoperatively revealed a radiopaque area at the site that underwent sinus floor elevation. Swelling of the sinus membrane was not observed (Figure 1c).

#### 3.1.2. Case 2

**Figure 2:**
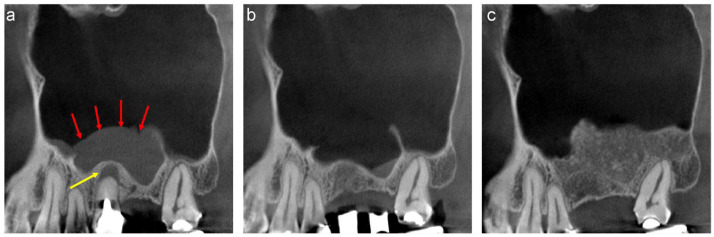
The mesiodistal section of CT images of the left maxillary sinus. (**a**) Well-defined, faintly radiopaque antral lesions (red arrows) and bone resorption at the palatal root apex of 26 (yellow arrow) are observed. (**b**) Almost all well-defined, faintly radiopaque antral lesions had disappeared four months after the extraction. (**c**) A radiopaque area is observed at the sinus floor elevation without swelling of the sinus membrane nine months postoperatively.

A 44-year-old female scheduled to undergo implant placement for the replacement of 27 was referred to our clinic in 2014 for sinus floor elevation owing to insufficient bone height. A well-defined, faintly radiopaque antral lesion was observed on the mesiodistal section of the CT image acquired at the first visit (Figure 2a, red arrows). A periapical lesion was observed at the palatal root apex of 26, indicating periapical disease (Figure 2a, yellow arrow); consequently, the tooth was extracted by the original dentist. The antral radiopaque lesion had disappeared almost completely four months after the extraction (Figure 2b). Thus, sinus floor elevation was performed using β-TCP granules alone at our clinic. CT image acquired nine months postoperatively revealed a radiopaque area at the site where sinus floor elevation was performed without swelling of the sinus membrane (Figure 2c).

### 3.2. Lymphangioma

Lymphangiomas are comparatively rare vascular tumours that arise in the maxillary sinus [14,15]. Small lymphangiomas are asymptomatic and do not require aggressive treatment. However, lymphangioma of the maxillary sinus can lead to the destruction of the adjacent tissue when it increases in size [16].

#### Case 3

**Figure 3:**
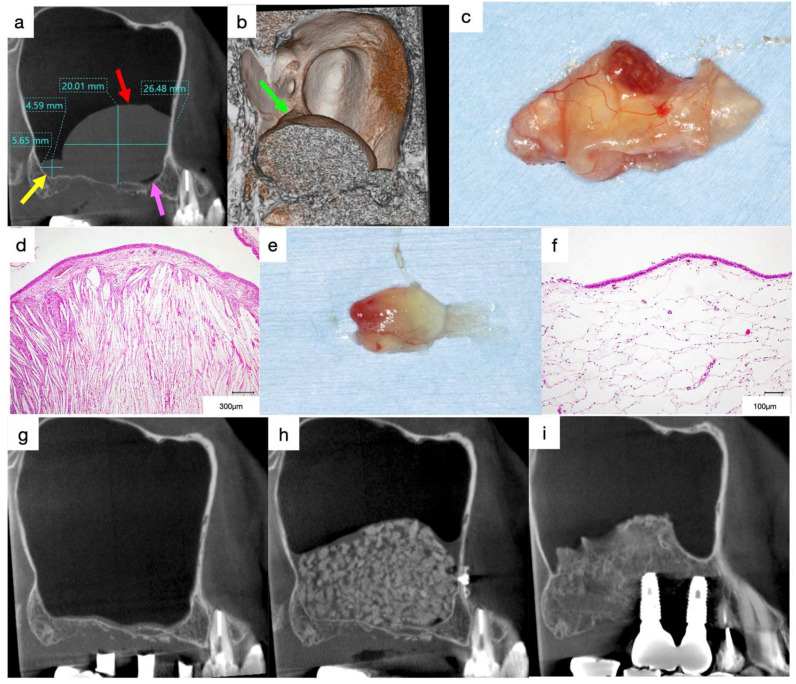
(**a**) Mesiodistal section of the CT image of the right maxillary sinus acquired at the first visit. Two well-defined, faintly radiopaque lesions are observed. The medial lesion is large (red arrow), and the distal lesion is small (yellow arrow). A radiolucent area is observed in the anteroinferior region of the large medial lesion (pink arrow). (**b**) Inside view of the mesiodistal section of the volume-rendered image. The base of the large lesion is located in the superior area of the lateral wall (green arrow). (**c**) The enlarged enucleated large medial lesion contains yellow serous fluid and abundant cholesterol crystals. (**d**) Histological specimen of a large medial lesion stained with haematoxylin and eosin (H&E). The outer surface of the lesion is lined with ciliated columnar epithelium and the inner surface is lined with fibrous connective tissue without epithelium. An abundance of needle-shaped spaces and many foreign-body giant cells were observed beneath the inner surface, which were diagnosed as AP. (**e**) The enucleated posterior small lesion is a whitish transparent soft mass. (**f**) Histological specimen of a small distal lesion (haematoxylin and eosin staining). Hyperplasia of the dilated lymphatic vessels beneath the ciliated columnar epithelial cells and slight infiltration of inflammatory cells can be observed. The patient was diagnosed with antral lymphangioma. (**g**) Mesiodistal section of the CT images acquired three months after enucleation. Swelling of the sinus membrane is not observed, suggesting that the function of the ciliated columnar epithelial cells was restored. (**h**) The sinus floor elevation was performed using β-TCP granules four months after the enucleation. (**i**) A radiopaque area can be observed at the site of sinus floor elevation two years after sinus floor elevation, and no recurrence of the lesions was confirmed.

A 64-year-old woman scheduled to undergo implant placement for the replacement of 15, 16, and 17, was referred to our clinic in 2021 for sinus floor elevation owing to insufficient bone height. Two well-defined, faintly radiopaque lesions were observed on the mesiodistal section of the CT image acquired at the first visit. The medial lesion was large (Figure 3a, red arrow), whereas the distal lesion was small (Figure 3a, yellow arrow). A radiolucent area was observed in the antero-inferior region of the large medial lesion (Figure 3a, pink arrow). The inner view of the mesiodistal section of the volume-rendering image revealed that the base of the large lesion was located in the superior area of the lateral wall (Figure 3b, green arrow). The radiolucent area appeared to be a space between the floor of the sinus and the large drooping medial lesion on the images (Figure 3a, pink arrow). The lesions were enucleated and examined histologically before sinus floor elevation.

The medial lesion was a pseudocyst containing yellow serous fluid and abundant cholesterol crystals (Figure 3c). Histological examination revealed that the outer surface of the lesion was lined with ciliated columnar epithelium, whereas the inner surface was lined with fibrous connective tissue without epithelium. An abundance of needle-shaped spaces (where cholesterol crystals had dissolved during tissue processing) and numerous foreign-body giant cells were observed beneath the inner surface, leading to a diagnosis of AP (Figure 3d). The small posterior lesion was a whitish transparent soft mass that was easily removed with a suction probe (Figure 3e). Histological examination revealed hyperplasia of dilated lymphatic vessels beneath the ciliated columnar epithelial cells and slight infiltration of inflammatory cells, leading to the diagnosis of antral lymphangioma (Figure 3f). The CT image acquired three months after the enucleation revealed no swelling of the sinus membrane, suggesting that the ciliated columnar epithelial cells had recuperated (Figure 3g). Maxillary sinus floor elevation was performed using β-TCP granules (Figure 3h) four months after enucleation, and two implants were placed by the previous dentist 11 months after sinus floor elevation. The CT image acquired 2 years after sinus floor elevation revealed no recurrence of the lesions, and the implants were functional (Figure 3i).

The small lesion was enucleated to enucleate the large lesion in this case. The lymphangioma would have presented as a small AP during maxillary sinus floor elevation if the large AP was not present. Sinusitis would have occurred due to the impairment of osteo-meatal complex patency if the size of the lymphangioma had increased during the maintenance period. Furthermore, enucleation of the lymphangioma would have become more difficult as the lesion moved upward owing to sinus floor elevation.

### 3.3. Gingival Cancer

The most critical differential diagnosis for AP is malignancy. Malignant tumour that are left untreated will grow infinitely and become life-threatening [17,18].

#### Case 4

**Figure 4:**
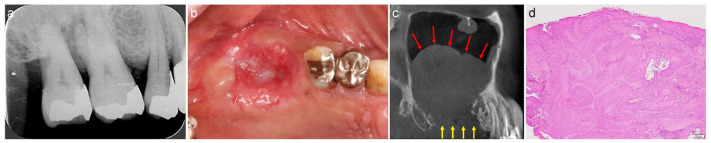
(**a**) Dental radiograph obtained by the original dentist. Slight bone resorption can be observed around the roots of 17 and 16. (**b**) Intraoral photograph acquired at the first visit. Reddish tissue is present in the extraction socket. Localized erosion can be observed at the palatal gingiva, whereas normal thick keratinised gingiva can be observed at the medial, buccal, and distal alveolar crests. (**c**) Mesiodistal section of CT images acquired at the first visit. A well-defined, faintly radiopaque lesion is observed in the maxillary sinus (red arrows). The floor of the maxillary sinus and alveolar bone surrounding 17 and 16 are fully absorbed (yellow arrows). (**d**) Histological specimen of the biopsy at the extraction socket (haematoxylin and eosin staining) indicating squamous cell carcinoma.

A 58-year-old male scheduled to undergo implant placement for the replacement of 16 and 17 was referred to our clinic in 2009 by his dentist for sinus floor elevation owing to insufficient bone height. A periapical radiograph acquired by the dentist revealed slight bone resorption around the roots of 17 and 16 (Figure 4a). The teeth were extracted, and curettage of the extraction socket was performed one month before the first visit to our clinic.

Reddish tissue was observed in the extraction socket in the intraoral photograph acquired at the first visit. Localized erosion of the palatal gingiva was observed; however, normal thick keratinised gingiva was observed at the medial, buccal, and distal alveolar crests (Figure 4b). The patient was asymptomatic. A well-defined, faintly radiopaque lesion was observed in the maxillary sinus on the mesiodistal section of the CT image acquired at the first visit (Figure 4c, red arrows). The floor of the maxillary sinus and the alveolar bone surrounding 17 and 16 were fully absorbed (Figure 4c, yellow arrows), leading to the diagnosis of suspected malignancy. The patient was referred to the Department of Oral and Maxillofacial Surgery of a University hospital. The lesion was diagnosed as squamous cell carcinoma of the gingiva (Figure 4d). Despite undergoing radical surgery and receiving radiation therapy and chemotherapy, the patient died due to metastasis 6 years after the first treatment.

## 4. Treatment of AP

Based on a previous publication by one of the authors, eighty-six APs in 52 maxillary sinuses were treated at the clinic [19]. Notably, in 25 (29.1%) of the 86 APs, the contents were viscous and challenging to aspirate. As a result, it was concluded that a two-stage surgery is the most reliable method for both AP enucleation and sinus floor elevation.

### 4.1. Patients and Informed Consent

Periodontitis or periapical diseases of the remaining teeth should be ruled out if well-defined, faintly radiopaque antral lesions are observed. Sinus floor elevation should be performed after treating the odontogenic diseases in such cases. The changes in the radiopaque lesions were evaluated using CT images acquired three months after the completion of dental treatment.

Destruction of the alveolar bone or maxillary sinus should be examined carefully in patients with well-defined, faintly radiopaque antral lesions without odontogenic diseases. Patients must be referred to the Department of Oral and Maxillofacial Surgery of general hospitals or universities for further examination if bone destruction is observed.

Patients presenting with well-defined, faintly radiopaque antral lesions without odontogenic disease or bone destruction are treated at our clinic. Lesions >8 mm in size or multiple lesions are enucleated and diagnosed histologically. Sinus floor elevation is performed without enucleation if the size of a single lesion is <8 mm. However, consent for CT follow-up and the possibility of further treatment should be obtained before sinus floor elevation.

### 4.2. Enucleation of APs

Azithromycin hydrate (500 mg) was administered 1 h before the surgery and once daily for 2 days postoperatively as prophylaxis against infection. All surgeries were performed under local anaesthesia with intravenous sedation using midazolam.

The frontal wall of the maxillary sinus is the best position for the placement of the bony window to survey the sinus cavity. Thus, a vertical incision was made in the vestibular sulcus, and a mucoperiosteal flap was elevated to expose the surgical site. A midcrestal incision was made to secure a sufficient field for the creation of a bone window in patients with large APs or thickened lateral walls. A bony window with a diameter of 10–13 mm was created in the frontal wall using a diamond round bur, and the sinus membrane was intentionally perforated.

**Figure 5:**
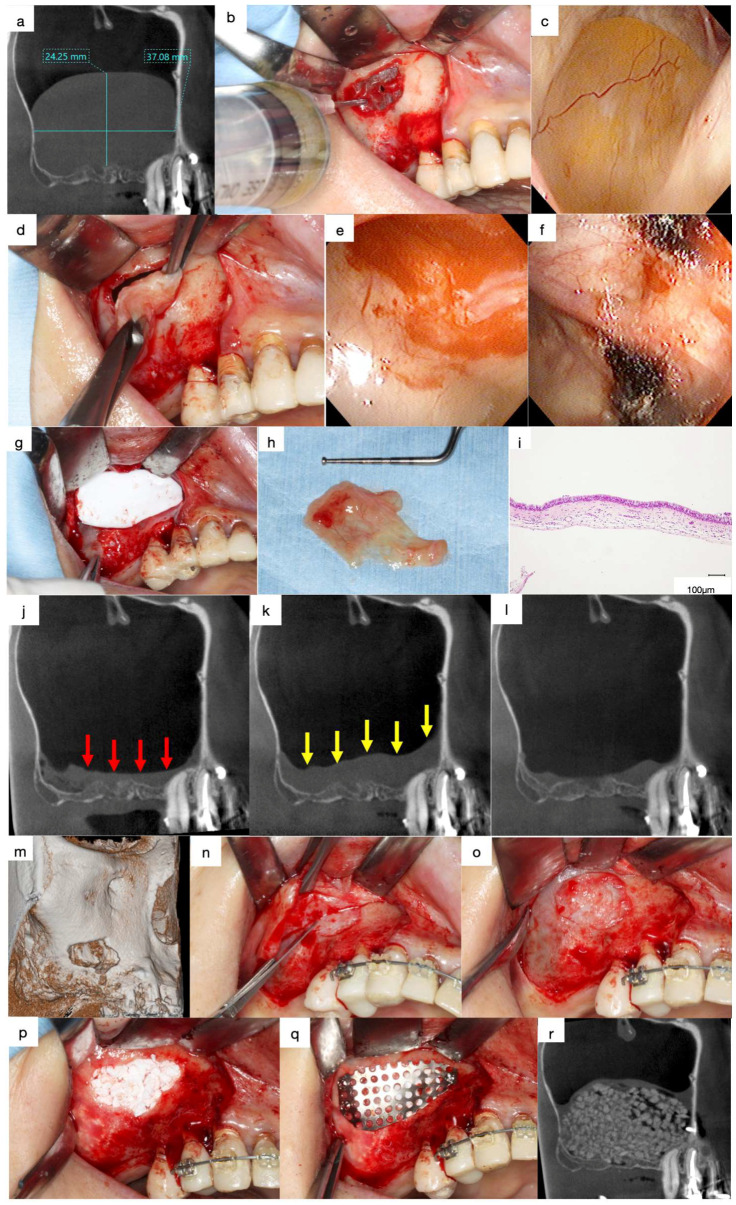
(**a**) Mesiodistal section of the CT images acquired at the first visit. A large, well-defined, and faint radiopaque lesion can be observed in the maxillary sinus. (**b**) Preliminary suction of the lesion contents was performed using a fine 18-gauge needle. (**c**) Endoscopic view of the antral pseudocyst (AP) after suction of the contents. The AP has reduced in size compared to that observed on the CT images during the preliminary section. (**d**) The surface of the AP was clamped with forceps and herniated through the bony window with gentle outwards traction. (**e**) Endoscopic view of the enucleated area. A contentious haemorrhage was observed at the base of the AP. (**f**) Oxidised regenerated cellulose was applied to stop bleeding. (**g**) The bony window is covered with a resorbable collagen membrane to reduce blood flow into the sinus cavity. (**h**) The AP was enucleated as a lump with a base. (**i**) Histological specimen of the enucleated AP (haematoxylin and eosin staining). The outer surface of the lesion was lined with ciliated columnar epithelium and the inner surface was lined with fibrous connective tissue without epithelium. (**j**) Mesiodistal section of the CT image obtained after enucleation. A flat, faintly radiopaque area can be observed, demonstrating an accumulation of physiological saline or blood at the bottom of the maxillary sinus (red arrows). (**k**) Swelling of the sinus membrane increased one-week following enucleation (yellow arrows). (**l**) Swelling of the sinus membrane decreased three months after enucleation. (**m**) Volume-rendering image obtained three months after enucleation. The bone defect persists in the bony window. (**n**) Dissection of the scar tissue between the sinus membrane and oral mucosa was necessary at the bone defect area to avoid perforation of the sinus cavity. (**o**) Scar tissue was observed in the bone defect area without perforation after the elevation of the mucoperiosteal flap. (**p**) β-TCP granules fill the space between the elevated periosteum and the exposed bone surface of the maxillary sinus. (**q**) The bony window was tightly covered with a titanium mesh plate and three titanium microscrews. (**r**) Mesiodistal section of the CT image obtained after sinus floor elevation. Sufficient radiopaque granules can be observed in the augmented area.

Preliminary suction of the contents was performed with a fine 18-gauge needle in cases with large APs (>15 mm) to reduce the volume to facilitate the clamping of the AP (Figure 5a,b). The surface of the AP was clamped with forceps and herniated through the bony window with gentle outwards traction without preserving the integrity of the periosteum underneath the AP (Figure 5c,d). The base of the AP, which appears as a sessile dome-shaped, well-defined, faintly radiopaque lesion, is usually present on the floor of the sinus cavity. In contrast, the AP appears as a round when the base of the AP is present at the lateral wall of the maxillary sinus (Figure 3a,b). The position of the base must be evaluated preoperatively as the AP, including the base, should be enucleated together.

The use of an endoscope is not mandatory; however, it aids in confirming the enucleation of APs and haemostasis through the small bony window. Haemostasis was performed using oxidised regenerated cellulose in cases with continuous haemorrhage (Figure 5e,f). The bony window was covered with a resorbable collagen membrane to reduce blood flow into the sinus cavity, and the mucoperiosteal flap was closed (Figure 5g).

All patients were informed of the possibility of postoperative intermittent epistaxis that may be observed for a few days. The patients were instructed not to blow their nose for the 2 weeks postoperatively to avoid wound dehiscence due to the momentary high pressure in the sinus cavity. Postoperative bleeding and infection were not observed in any of the patients, and all sutures were removed eight days following surgery.

**Figure 6:**
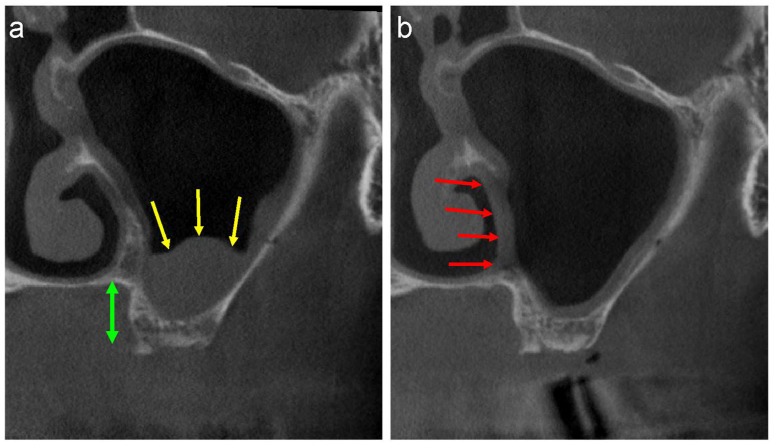
(**a**) The buccolingual section of the CT image shows well-defined, faintly radiopaque antral lesions (yellow arrows), and the distance between the alveolar crest and bottom of the nasal cavity is short (green up-down arrow). (**b**) A bone defect in the medial wall is observed following the removal of the lesion by the ENT surgeon (red arrows).

Endoscopic intranasal sinus surgery, a method commonly used to remove maxillary sinus cysts, is another surgical procedure commonly used for the enucleation of APs [20,21]. However, this surgery is usually performed by an ENT surgeon under general anaesthesia. The medial wall of the maxillary sinus must be removed when the surgeon approaches the inferior nasal meatus in this approach. Sinus floor elevation is difficult to perform in cases where the distance between the alveolar crest and the floor of the nasal cavity is short (Figure 6a, green arrows) owing to the bone defect in the medial wall (Figure 6b, red arrows). Therefore, a preoperative discussion between the ENT and oral surgeons is necessary.

### 4.3. Histopathological Findings of APs

The characteristic histological findings of AP include the absence of a lining epithelium at the inner surface of the cystic lesion and fluid accumulation within the fibrous connective tissue beneath the outer surface of the ciliate pseudostratified epithelium (Figure 5h,i). Cysts are characterized by the presence of lining epithelium on the inner surface of cystic lesions; thus, AP has been distinguished from cysts using the term “pseudo”.

The lesion is diagnosed as a mucous retention cyst in cases where the inner surface of the cystic lesion in the maxillary sinus is lined with ductal epithelium. The lesion is diagnosed as a mucocele in cases where the inner surface is lined with ciliate pseudostratified epithelium [22,23,24]. Mucoceles of the maxillary sinus differ from those observed in the oral cavity and can lead to bone destruction [7,25].

### 4.4. Lateral Approach after Enucleation

Swelling of the sinus membrane is observed due to traumatic stimulation one week post-surgery (Figure 5j,k), which diminishes three months later (Figure 5l). The ciliated columnar epithelium cells at the base of the AP are removed during the enucleation, and the periosteum underneath the AP is exposed. Hence, the decrease in the swelling of the sinus membrane indicates the regeneration of the columnar epithelial cells and restoration of the ciliary motility. Thus, sinus floor elevation can be performed four months following enucleation of APs.

Bone defects in the bony window persist for four months following the enucleation of APs (Figure 5m). Therefore, dissection of the scar tissues between the sinus membrane and oral mucosa must be performed in the area with the bone defect during the elevation of the mucoperiosteal flap for sinus floor elevation using the lateral approach (Figure 5n,o). The periosteum underneath the sinus membrane is elevated after enlarging the bony window, and the β-TCP granules are filled in the space between the elevated periosteum and the exposed bone surface of the maxillary sinus (Figure 5p). The bony window is covered tightly with a titanium mesh plate and three titanium micro screws to avoid the migration of the β-TCP granules due to the postoperative sealing of the sinus membrane (Figure 5q,r) [5].

## 5. Chronological CT Evaluation after the Sinus Floor Elevation Using β-TCP Granules Alone

### 5.1. Before the Placement of Implants

**Figure 7:**
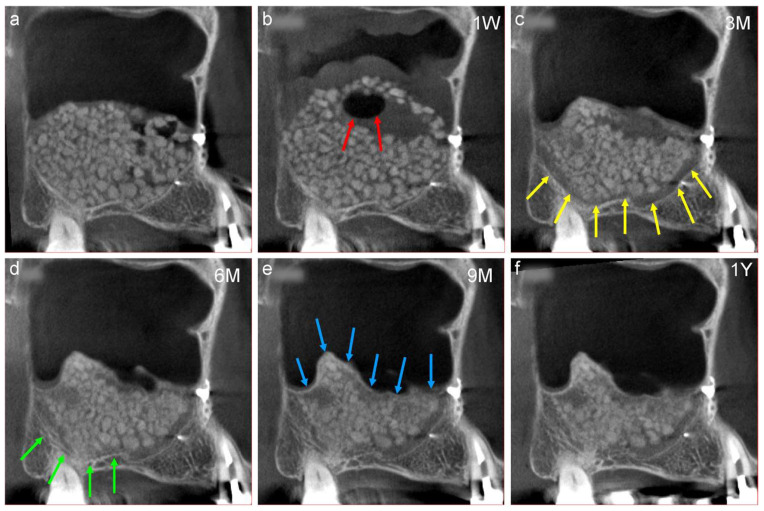
(**a**) Mesiodistal section of the CT image obtained after sinus floor elevation. Sufficient β-TCP granules are observed at the augmented area. (**b**) One week after surgery, the β-TCP granules moved upward owing to postoperative swelling of the sinus membrane. An air around β-TCP granules has accumulated at the upper portion of the augmented area (red arrows). 1W; one week follow-up (**c**) Swelling of the sinus membrane disappeared spontaneously three months postoperatively, and a belt-shaped radiolucency (grey zone) is observed at the interface between the β-TCP granules and the surface of the maxillary sinus (yellow arrows). 3M; three months follow-up (**d**) The radiopacity of the original cortical bone decreased six months postoperatively, and that of the grey zone increased (green arrows). 6M; six months follow-up (**e**) A complete radiopaque line (white line) is observed at the newly formed bottom of the maxillary sinus (blue arrows) nine months postoperatively. 9M; nine months follow-up (**f**) The white line becomes clear 12 months postoperatively; however, the β-TCP granules in the central area retained their shape. 1Y; one year follow-up.

Only β-TCP granules are used as bone material for sinus floor elevations at our clinic (Figure 7a) as they can transform into actual bone and are not retained as foreign bodies around the implants. Furthermore, the transformation of the β-TCP granules can be evaluated using CT, and the best period for the placement of implants can be determined according to the individual biological reaction.

The representative chronological CT images of a 58-year-old female are described below.

1. The β-TCP granules moved upward owing to postoperative swelling of the sinus membrane one week postoperatively. These movements of bone materials determine the final shape of the augmented area (Figure 7b,f). As the weight of the air surrounding the β-TCP granules is comparatively low, it accumulated in the upper portion of the augmented area (Figure 7b, red arrows).
2. Swelling of the sinus membrane resolved spontaneously three months postoperatively, and a belt-shaped radiolucency (grey zone) can be observed at the interface between the β-TCP granules and the surface of the maxillary sinus (Figure 7c, yellow arrows). This grey zone signifies that the osteoclasts have resorbed the β-TCP granules and the original bone [26], which is an important sign of bone regeneration.
3. The radiopacity of the original cortical bone decreases six months postoperatively, whereas that of the grey zone increases (Figure 7d, green arrows).
4. A completely radiopaque line (white line) can be observed at the newly formed floor of the maxillary sinus nine months postoperatively (Figure 7e, blue arrows). This white line represents the newly formed bone derived from the periosteum underneath the elevated sinus membrane. The immature bone tissue in the augmented area did not move during drilling for the placement of implants. Thus, the completely white line could be considered an indicator of the implant placement period. There are great differences in the individual ability for bone transformation, so recommending the same period for the placement of implants in a uniform manner may be unsuitable. The β-TCP granules in the central area retained their shape 12 months postoperatively (Figure 7f). Thus, the transformation of the β-TCP granules into bone occurs according to their osseous conductive ability from the bone surface of the maxillary sinus.

### 5.2. After the Placement of Implants

**Figure 8:**
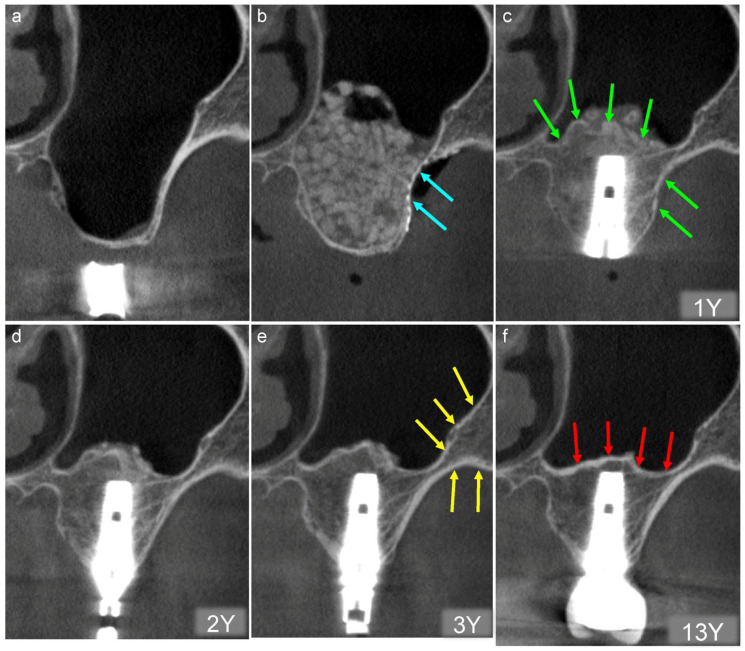
(**a**) Buccolingual section of the CT image obtained before sinus floor elevation. Bone height is <1 mm. (**b**) CT image acquired on the day of the surgery. The β augmented area is filled with TCP granules, and the bony window is covered tightly with a titanium mesh plate and microscrews (blue arrows). (**c**) Tapered-type implants were placed one year postoperatively, and initial fixation was achieved. A clear white line and radiopaque line resembling the cortical bone can be observed at the newly formed bottom of the maxillary sinus and bony window, respectively (green arrows). 1Y; one year follow-up (**d**) The radiopacity of the augmented area is divided into two types two years postoperatively: cortical and cancerous. 2Y; two years follow-up (**e**) The radiopacity of the augmented area shows a density similar to that of the original bone (yellow arrows) three years postoperatively. 3Y; three years follow-up (**f**) The bottom line of the maxillary sinus decreased slightly because of pneumatization 13 years postoperatively (red arrows). 13Y; thirteen years follow-up.

Representative chronological CT images of a 66-year-old female are shown below. In this case, sinus floor elevation was performed using β-TCP granules alone (Figure 8a,b), and the bony window was covered tightly with titanium mesh plate and microscrews (Figure 8b, blue arrows).

1. Tapered-type implants were placed to achieve initial fixation one year after sinus floor elevation. Small particles of β-TCP granules can be observed around the implant. However, a clear white line and radiopaque line similar to the cortical bone can be observed at the newly formed bottom of the maxillary sinus and bony window, respectively (Figure 8c, green arrows).
2. The radiopacity of the augmented area can be divided into two types two years after sinus floor elevation: cortical and cancerous bone. Tiny particles of β-TCP granules can be observed around the implant (Figure 8d).
3. The β-TCP granules have disappeared three years after sinus floor elevation, and the augmented area shows cortical and cancerous bone-like radiopacity. The radiopacity of the augmented area is similar to that of the original bone (Figure 8e, yellow arrows). This suggests that new bone tissue has likely formed in the augmented area, mirroring the density of the original bone.
4. The radiopacity of the augmented area remained almost unchanged compared with that at three years postoperatively 13 years after sinus floor elevation. Thus, an active bone transformation of the β-TCP granules continues for three years after sinus floor elevation. This results in the regeneration of the critical and cancerous bone. However, the line at the bottom of the maxillary sinus descended slightly due to pneumatization (Figure 8f, red arrows), suggesting that the newly formed bone is natural bone.

## 6. Sinus Floor Elevation Using Non-Absorbable Artificial Bone

Non-absorbable artificial bone has been used for sinus floor elevation, and favourable results have been reported across the world [27,28]. The most important advantage of using non-absorbable artificial bones is that sufficient strength for the placement of implants can be attained in the early period after sinus floor elevation. However, osseointegration occurs only with the newly formed bone around the non-absorbable artificial bones, not the artificial bones. Furthermore, the non-absorbable artificial bone is retained as a foreign body around the implants, unlike β-TCP granules. Hence, sinusitis can spread through artificial bones if infection, such as peri-implantitis, spreads around the implants.

A 69-year-old male was referred to our clinic in 2016 with chief complaints of headache, nasal congestion, and postnasal discharge. He had undergone implant treatment with sinus floor elevation using Bio-Oss 10 years prior. The implants replacing 17 and 16 had been shed two months before the first visit, and the patient had developed sinusitis.

**Figure 9:**
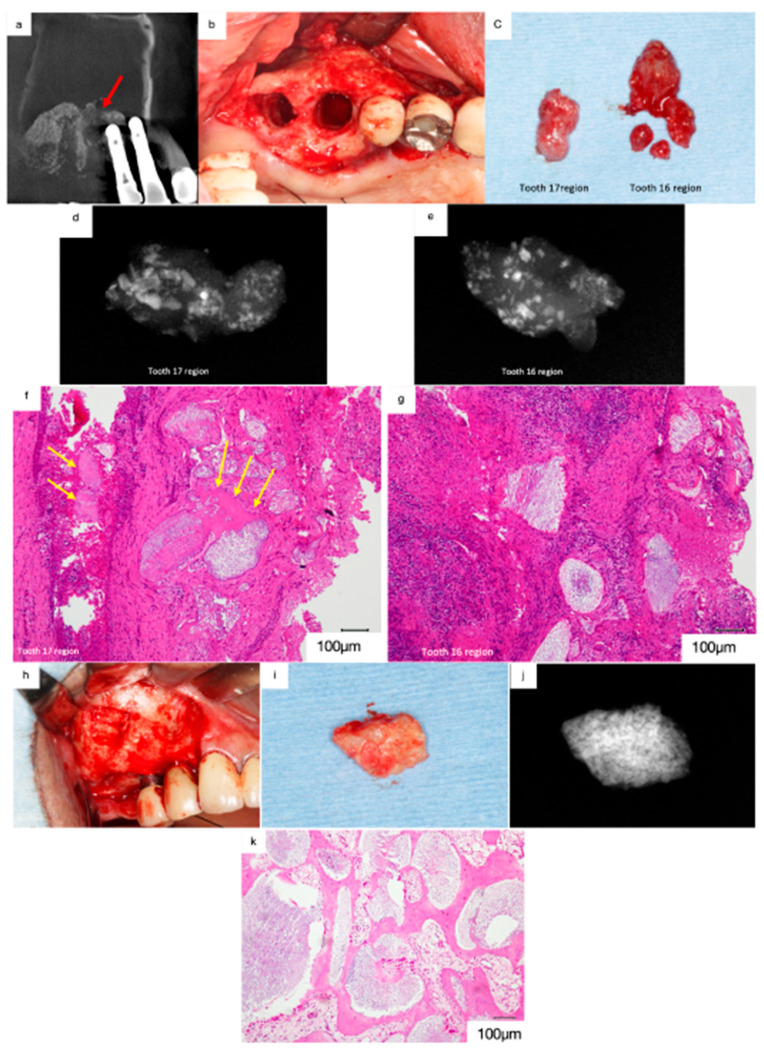
(**a**) Mediodistal section of the CT image acquired at the first visit. Prominent swelling of the sinus membrane can be observed, and no pneumatic space is present in the maxillary sinus. Artificial bone is absent at the floor of the maxillary sinus near 16 (red arrow). (**b**) The soft tissues surrounding 17 and 16 were removed. (**c**) Artificial bone granules were observed in the removed soft tissues. (**d**) Radiograph of the removed soft tissues surrounding 17. Radiopaque particles of various sizes were observed in the soft tissue. (**e**) Radiopaque particles of various sizes can be observed in the soft tissue surrounding 16. (**f**) Histological decalcified specimen of the soft tissue surrounding 17 (haematoxylin and eosin staining). Bio-Oss granules can be observed among the fibrous connective tissues with moderate infiltration of inflammatory cells, and only small bone tissues remain beside the Bio-Oss granules (yellow arrows). (**g**) Severe infiltration of inflammatory cells was observed in the fibrous connective tissue surrounding 16, and no live bone remains around the Bio-Oss granules. (**h**) Bone biopsy performed on the lateral wall. (**i**) White particles are grossly embedded in the harvested hard tissue. (**j**) Radiograph of the specimen showing dense radiopaque particles in the harvested tissue. (**k**) The demineralised histological specimen (H.E. staining) shows that newly formed live bone is present adjacent to the Bio-Oss granules, and many foreign body giant cells can be observed around the Bio-Oss granules.

The mediodistal section of the CT image acquired at the first visit revealed prominent swelling of the sinus membrane. Pneumatic space was not observed in the maxillary sinus (Figure 9a). Radiolucent areas containing radiopaque particles were observed in the regions surrounding 17 and 16. Furthermore, artificial bone was absent at the floor of the maxillary sinus in the region surrounding 16, suggesting that the maxillary sinus was interconnected with the socket of the lost implant (Figure 9a, red arrows).

The removal of the soft tissue surrounding 17 and 16 revealed artificial bone granules (Figure 9b,c). Radiographs acquired subsequently revealed radiopaque particles of varying sizes within the tissues (Figure 9d,e). The demineralised histological specimen (H&E staining) revealed the presence of Bio-Oss granules in the fibrous connective tissues (Figure 9f,g). Moderate infiltration of inflammatory cells was observed in the fibrous connective tissues in the regions surrounding 17, and only small bone tissues were observed beside the Bio-Oss granules (Figure 9f, yellow arrows). In contrast, severe infiltration of inflammatory cells was observed in the regions surrounding 16, and no live bone was observed around the Bio-Oss granules (Figure 9g). These findings suggest that peri-implantitis disrupted osseointegration and that Bio-Oss granules were isolated from the newly formed bone tissue. Furthermore, the infection spread to the maxillary sinus through the Bio-Oss granules in the regions surrounding 16, leading to sinusitis.

A bone biopsy was performed on the lateral wall after receiving consent from the patient (Figure 9h,i). Radiographs of the harvested specimen revealed dense radiopaque particles (Figure 9j). The demineralised histological specimen (H&E staining) revealed the presence of newly formed live bone adjacent to the Bio-Oss granules (Figure 9k). Furthermore, numerous foreign body giant cells were observed around the Bio-Oss granules, suggesting that the biological reaction to the Bio-Oss granules had persisted for 10 years postoperatively. Thus, non-absorbable bone materials are associated with a risk of inducing sinusitis in patients with peri-implantitis when used as bone material for sinus floor elevation.

## 7. Discussion

The most important aspect of implant treatment is the conservation of osseointegration. Simensen reported that patients expect their implants to be retained for >10 years after treatment [29]. Hence, implant treatment can be considered successful if the implants function uneventfully for >5 years after treatment when combined with maxillary sinus floor elevation [30]. However, several unknown phenomena are associated with the biological reactions occurring in response to foreign bodies such as implants or artificial bones. Therefore, the risk factors for bone destruction must be eliminated to ensure osseointegration. Swelling of the sinus membrane should be resolved preoperatively to avoid the occurrence of sinusitis or the requirement for further surgical intervention in patients scheduled to undergo sinus floor elevation.

Well-defined, faintly radiopaque antral lesions were observed incidentally on the CT images of patients requiring maxillary sinus floor elevation. Diseases of the remaining adjacent teeth should be ruled out to determine the secondary cause of swelling of the maxillary sinus membrane. Sinusitis can occur due to the infection if sinus floor elevation is performed without treating the adjacent teeth. The presence or absence of bone destruction around the well-defined, faintly radiopaque antral lesions should be carefully examined on CT images. The diagnosis of odontogenic cysts, mucoceles, or tumours should be considered if bone destruction is observed. However, bone destruction is not always observed in patients with early stage lesions. Thus, long-term observation of the lesions using CT images is mandatory if sinus floor elevation is performed without the enucleation of the well-defined, faintly radiopaque antral lesions. However, enucleation of the lesion becomes more difficult as the position of the lesion moves upward due to sinus floor elevation [2]. The bone material used during sinus floor elevation influences the postoperative observation of lesions. The transformation of the β-TCP granules into natural bone tissue can be evaluated easily using CT images. However, they remain close to the lesions when non-absorbable artificial bone particles are used for sinus floor elevation. Moreover, the lesions are influenced by various biological reactions to the foreign body. Thus, β-TCP granules seem to be more advantageous than non-absorbable artificial bone particles in cases wherein well-defined, faintly radiopaque antral lesions are retained.

The suction of the contents is not effective for the treatment of AP, as the contents will accumulate again without any symptoms [31]. Moreover, approximately 30% of the contents of APs are viscous and difficult to suction, according to our clinical data [19]. Furthermore, the contents would pour into the augmented area when the periosteum is ruptured even if the APs are enucleated during sinus floor elevation. The augmented area could become contaminated if the contents contain cholesterol crystals, which could hinder bone formation. However, the content of APs is indiscernible even on CT images obtained preoperatively. Consequently, sinus floor elevation should be performed 4 months after enucleation and histological diagnosis of the lesions. This time lag seems to ensure the success of sinus floor elevation.

## 8. Conclusions

The diagnosis of well-defined, faintly radiopaque antral lesions is necessary in patients undergoing sinus floor elevation. Sinus floor elevation should be performed four months after enucleation in patients with lesions histopathologically diagnosed as AP. Long-term CT evaluation should be performed in cases wherein sinus floor elevation is performed without enucleation of the lesion.
